# Long-term Clinical Outcomes After Single- Versus Double-Bundle ACL Reconstruction: A Matched-Pair Analysis From the SANTI Study Group

**DOI:** 10.1177/23259671251364262

**Published:** 2025-09-15

**Authors:** Archie Todd-Hems, Alessando Carrozzo, Graeme P. Hopper, Jae-Sung An, Mohammed Lahsika, Giancarlo Giurazza, Thais Dutra Vieira, Bertrand Sonnery-Cottet

**Affiliations:** * NHS Lanarkshire University Hospitals, Glasgow, Scotland, United Kingdom; † Centre Orthopédique Santy, Lyon, France; Hôpital Privé Jean Mermoz, Ramsay-Générale de Santé, Lyon, France; Investigation performed at Centre Orthopédique Santy, Lyon, France

**Keywords:** knee, ACL reconstruction, double-bundle reconstruction, graft survivorship

## Abstract

**Background:**

Anterior cruciate ligament (ACL) reconstruction (ACLR) techniques have evolved toward more “anatomic” approaches, including the double-bundle (DB) method, which theoretically offers superior rotational control compared with single-bundle (SB) reconstruction. However, few studies have investigated comparative outcomes at mid- to long-term follow-up.

**Purpose/Hypothesis:**

The purpose of this study was to compare the long-term clinical outcomes of SB and DB ACLR. It was hypothesized that both techniques would yield similar clinical results over an extended follow-up period.

**Study Design:**

Cohort study; Level of evidence, 3.

**Methods:**

Primary ACLR cases using either a DB or SB hamstring autograft technique registered in the Santy database from 2003 through 2008 were eligible. Propensity score matching was used to adjust for covariates such as age, sex, body mass index, meniscal status, and sports participation. Clinical evaluations—graft survival, side-to-side laxity measurements, and reoperation rates—were analyzed using the Kaplan-Meier method and Cox proportional hazards model.

**Results:**

A total of 396 patients—198 in each group—were included, at a mean follow-up of 14.7 ± 0.6 years. In total, 40 patients (10.1%) experienced graft rupture, with no statistically significant difference between the 22 patients (11.1%) in the SB group and 18 patients (9.1%) in the DB group (*P* = .52). Reoperation rates, including for cyclops syndrome, arthrofibrosis, or meniscal pathology, were also comparable between groups. There was no statistically significant difference in anterior laxity measurements at 1 year (*P* = .11). Kaplan-Meier and Cox proportional hazards analyses showed no association between reconstruction technique and risk of graft failure (HR, 0.857; 95% CI, 0.457-1.609; *P* = .63).

**Conclusion:**

This study showed that over a mean follow-up of >14 years, the SB and DB ACLR techniques using hamstring autograft showed similar clinical results in terms of graft survival, reoperation rates, and stability. These results suggest that both techniques offer comparable efficacy in the long term.

Over the past decades, anterior cruciate ligament (ACL) reconstruction (ACLR) has undergone a remarkable evolution, transitioning from open surgery and rudimentary graft fixation techniques in the early 20th century to highly refined arthroscopic procedures by the 1990s. By the early 2000s, abundant clinical data clarified numerous technical and anatomic questions, and arthroscopic ACLR became a routine procedure performed tens of thousands of times annually in both the United States and Europe.^19^ Nevertheless, concerns remained over long-term failure rates and residual pivot shift, particularly when the reconstruction failed to adequately control rotational instability.^15^ In response, surgical techniques have evolved toward more anatomic approaches, including the double-bundle (DB) method, which reconstructs both the anteromedial and posterolateral bundles to more closely mimic native knee biomechanics.^1,4,12,23^ Although multiple biomechanical studies have suggested that DB ACLR may provide superior rotational control compared with the conventional single-bundle (SB) technique, it remains unclear whether these potential biomechanical advantages consistently translate into better clinical outcomes.^12,23^ Moreover, issues such as increased surgical complexity, graft fixation challenges, and the risk of tunnel malposition fueled the debate. As a result, there has been a decline in the use of this technique over the years, and only a small percentage of surgeons still perform this procedure.^21^

To date, several investigations have compared clinical outcomes of SB and DB techniques, yet no definitive consensus has been established.^2,3,8,10,31^ Moreover, relatively few studies provide mid- to long-term follow-up data.^2,3,8,10,31^ Nonetheless, some reports indicate that DB procedures may result in improved range of motion and knee stability, fewer graft failures, and reduced rates of osteoarthritis (OA) compared with SB reconstruction.^10,17,23,29^

Because few studies have investigated comparative outcomes at mid- to long-term follow-up, the aim of this study was to evaluate long-term clinical outcomes after SB versus DB ACLR. It was hypothesized that the 2 techniques would yield similar clinical results.

## Methods

### Study Design and Participants

Institutional review board approval (COS-RGDS-2021-06-008-SONNERYCOTTET-B) was granted for this retrospective, nonrandomized, matched-paired comparative study, and all patients provided informed consent. Established in 2003, this database, following a register-based methodology, was designed to prospectively collect data on all cruciate ligament surgeries performed by the senior surgeon (B.S.C.). From this database, we selected the period during which the senior surgeon performed DB ACLR. Indications for a particular type of ACLR were based on patient factors, patient choice, and the evolving indications for specific techniques during the study period. Within this time frame, we matched patients who underwent DB ACLR with those who underwent SB ACLR using a hamstring autograft. Between October 2003 and November 2008, data were prospectively collected on patients who underwent ACLR. Patients were excluded if they underwent revision surgery, major concomitant procedures (including multiligament reconstructions and osteotomy), combined lateral extra-articular procedures, or primary ACLR using an autograft other than hamstring. Patient information was recorded through a standardized postoperative questionnaire completed by the surgeon, documenting preoperative and perioperative details. The database has been continuously updated with follow-up data collected at a minimum of 2 years postoperatively for eligible patients.

### Surgical Technique

All surgical procedures were performed by the senior author (B.S.C.). All patients were positioned supine with the knee flexed to 90°. A lateral support, foot roll, and tourniquet were utilized. Arthroscopic inspection of the knee and meniscal surgery were performed before the ACLR.

### Double-Bundle ACLR

**Figure 1.**
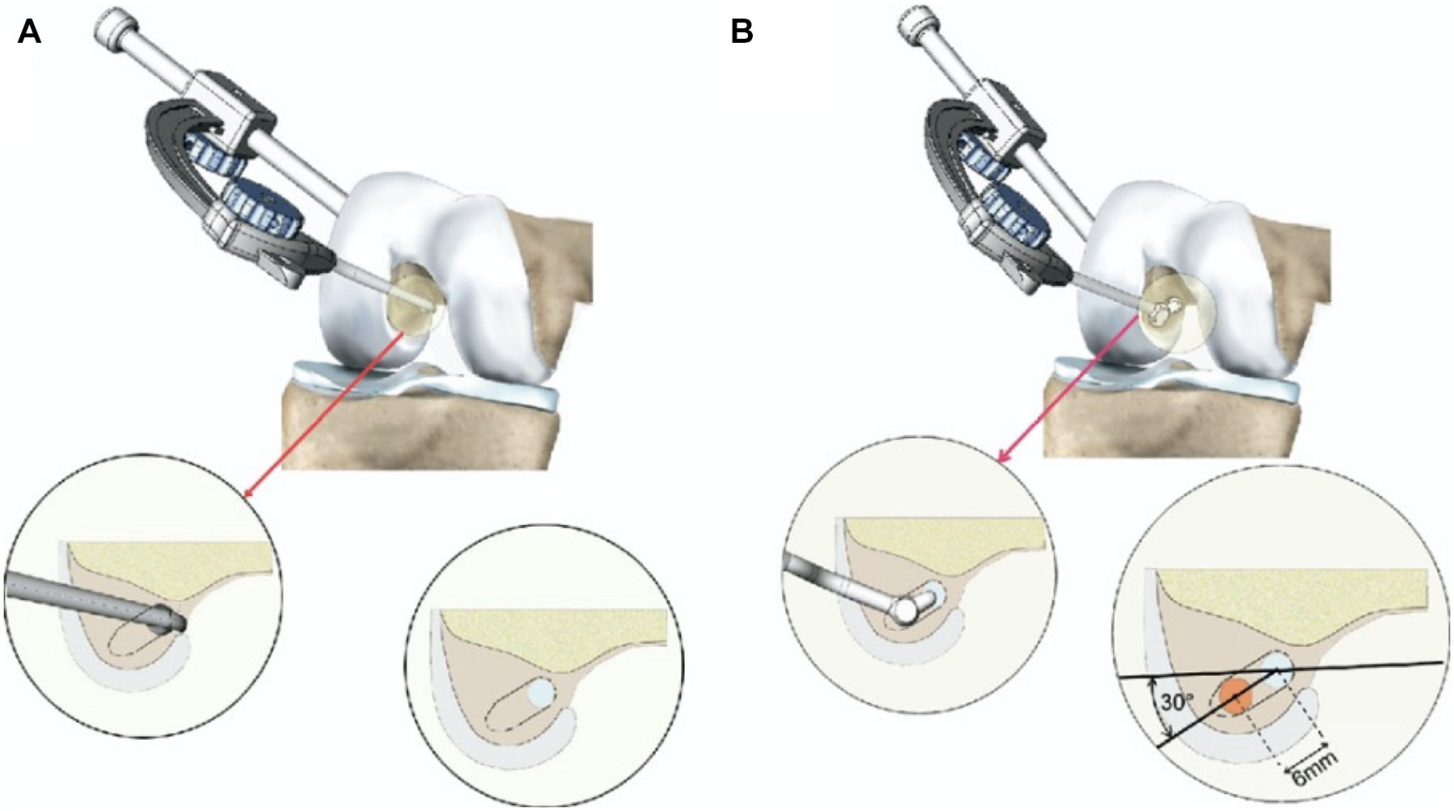
(A) The anteromedial tunnel is drilled from outside-in by use of an “out-in” guide. (B) The posterolateral tunnel is drilled through the same incision by use of a specific guide with its tip engaged in the anteromedial tunnel and allowing placement of a guide wire 6 mm distal and 30° posterior to the anteromedial tunnel.

The semitendinosus (ST) and gracilis tendons were harvested using an open-ended tendon stripper. The tibial insertion was preserved to improve fixation and vascularity.^17,29^ The femoral tunnels were drilled in an outside-in fashion. The ST tendons were doubled to form the anteromedial bundle, and the gracilis tendons were tripled to form the posterolateral bundle. The graft was fixed on both sides with bioabsorbable screws (Bio-Interference screw; Arthrex). The posterolateral bundle was tensioned and fixed in extension and the anteromedial bundle with the knee flexed at 45° (Figure 1).^23^

### Single-Bundle ACLR

**Figure 2.**
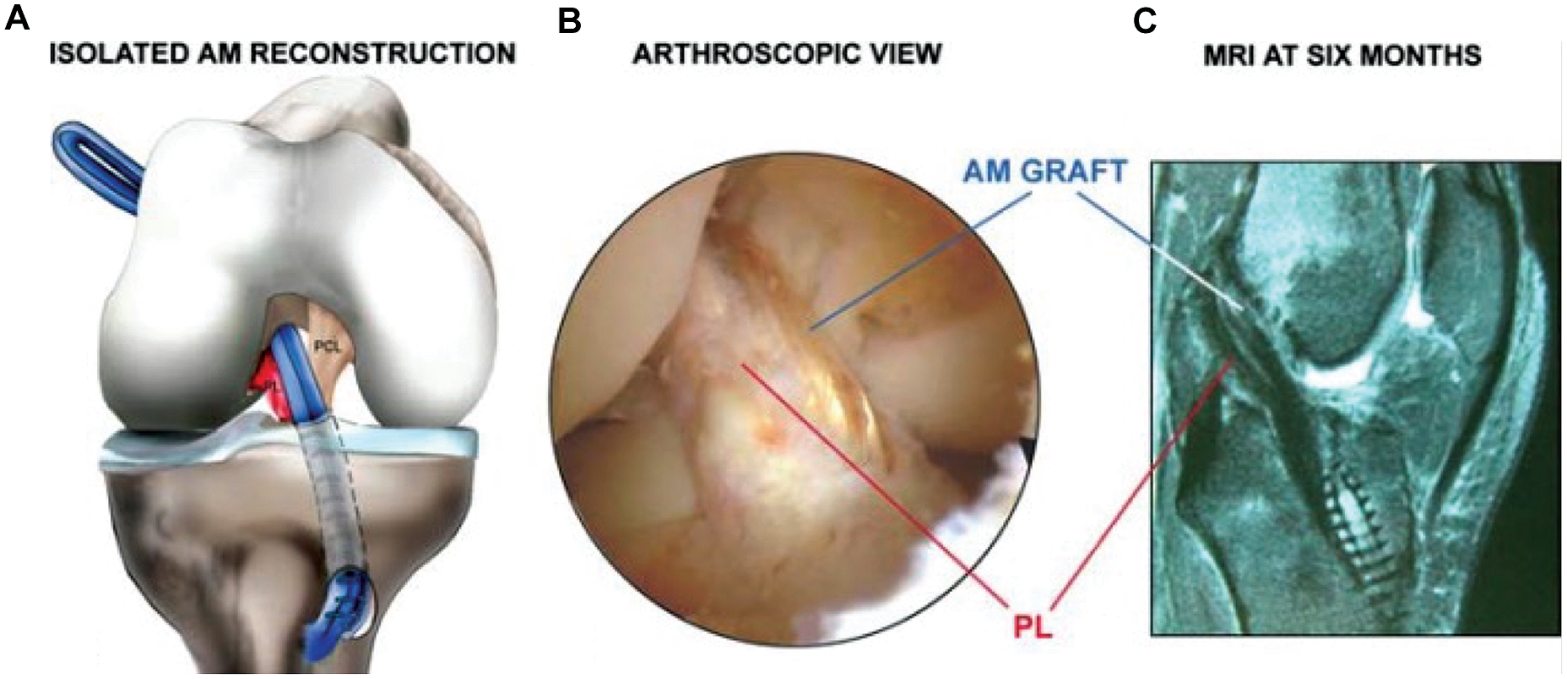
(A) The hamstring graft is routed from the tibia to the femur. The graft’s tibial insertion is preserved to improve further tibial fixation. (B) Arthroscopic view. (C) Magnetic resonance imaging (MRI) scan of the isolated anteromedial (AM) bundle reconstruction with the intact posterolateral bundle (PL) and the AM graft demonstrated.

The ST and gracilis tendons were harvested using an open-ended tendon stripper. The tibial insertion was preserved to improve fixation and vascularity.^17,30^ The femoral tunnel was drilled in an outside-in fashion. Tendons were quadrupled and then fixed on both sides with bioabsorbable screws (Bio-Interference screw). The graft was tensioned and fixed on the femoral side with the knee flexed at 30° (Figure 2).^25^

### Postoperative Rehabilitation

All patients participated in the same rehabilitation program regardless of the surgical technique. This consisted of brace-free, immediate full weightbearing and progressive range of motion exercises. Range of motion was restricted to 0° to 90° for 6 weeks for patients who underwent meniscal repair. Early rehabilitation was focused on maintaining full extension and quadriceps activation exercises. Return to sport was allowed at 4 months for nonpivoting sports, 6 months for pivoting noncontact sports, and 8 to 9 months for pivoting contact sports.

### Follow-up and Data Collection

All patients underwent follow-up by a fellowship-trained orthopaedic surgeon or a fellowship-trained sports medicine physician at 3 and 6 weeks and 3, 6, 12, and 24 months. Physical examination and side-to-side measurements with a Rolimeter (Aircast Europa) were performed to assess the stability of the operated knee.

Final follow-up for each patient was defined by the last patient follow-up recorded in prospectively collected data from the Santy database. Patient notes were reviewed by an investigator, independent of the primary surgeon, to determine if the patient had sustained a further ipsilateral knee injury or contralateral knee injury, had undergone any reoperations, or had any complications after the index procedure. ACL graft failure was diagnosed on magnetic resonance imaging examination in patients who had sustained a further injury or had symptoms suggestive of instability. Key patient characteristics and additional secondary surgery were also documented.

### Propensity Score Matching

Propensity score matching was undertaken to mitigate the effects of any possible treatment selection bias and allow the creation of 2 groups (DB group and SB group), in which confounding factors were balanced. A propensity score was determined for each patient based on the following criteria: age at the time of surgery (categorized as <20, 20-30, or >30 years), sex, body mass index (BMI) (World Health Organization categories),^16^ meniscal status, and participation in pivoting and contact sports. After this, patients who underwent DB ACLR were individually matched with a patient from the SB ACLR group according to the closest corresponding propensity score (Mahalanobis distance).^13^ After matching, the standardized difference was calculated as the difference in proportions of each level of the covariate or means divided by the pooled standard deviation. If the absolute magnitude of the standardized bias was greater than a prespecified threshold value (0.25), then imbalance for the covariate was indicated.

### Statistical Analysis

Descriptive data analysis was conducted depending on the nature of the considered criteria. For qualitative data, this included the number of filled and missing data and, for each modality, the frequency and percentage (referring to filled data). Proportions were estimated with their exact 95% confidence intervals when appropriate. Comparisons of data were made using the chi-square or Fisher exact test, according to the expected values under the assumption of independence. For quantitative data, this included the number of filled and missing data, arithmetic mean, standard deviation, median, first and third quartiles, minimum, and maximum. Comparisons of data were made using a Student *t* test or Mann-Whitney-Wilcoxon test, depending on the distribution of the variable of interest. The risk of occurrence of graft failure was described in terms of probability of occurrence and confidence interval using the Kaplan-Meier method. The different groups were compared using the log-rank test. For analysis of the occurrence of graft failure, survival analysis was performed using a Cox proportional hazards model considering different adjustment factors. The Breslow method was used to account for tied times. The Cox model expresses the instantaneous risk of an event as a function of time and covariates. The Cox model considers the follow-up period for each patient in addition to the occurrence of the event; therefore, the risk of event estimation is unbiased. Specifically, in this study, the principle of the Cox model was to associate the date of the occurrence of an event (graft failure or secondary surgery) with a type of treatment (SB ACLR vs DB ACLR) completely independent of the overall follow-up duration. Statistical significance was set at a *P* value <.05. All analyses were performed with SAS for Windows (Version 9.4; SAS Institute Inc).

## Results

### Patient and Clinical Characteristics

**Figure 3.**
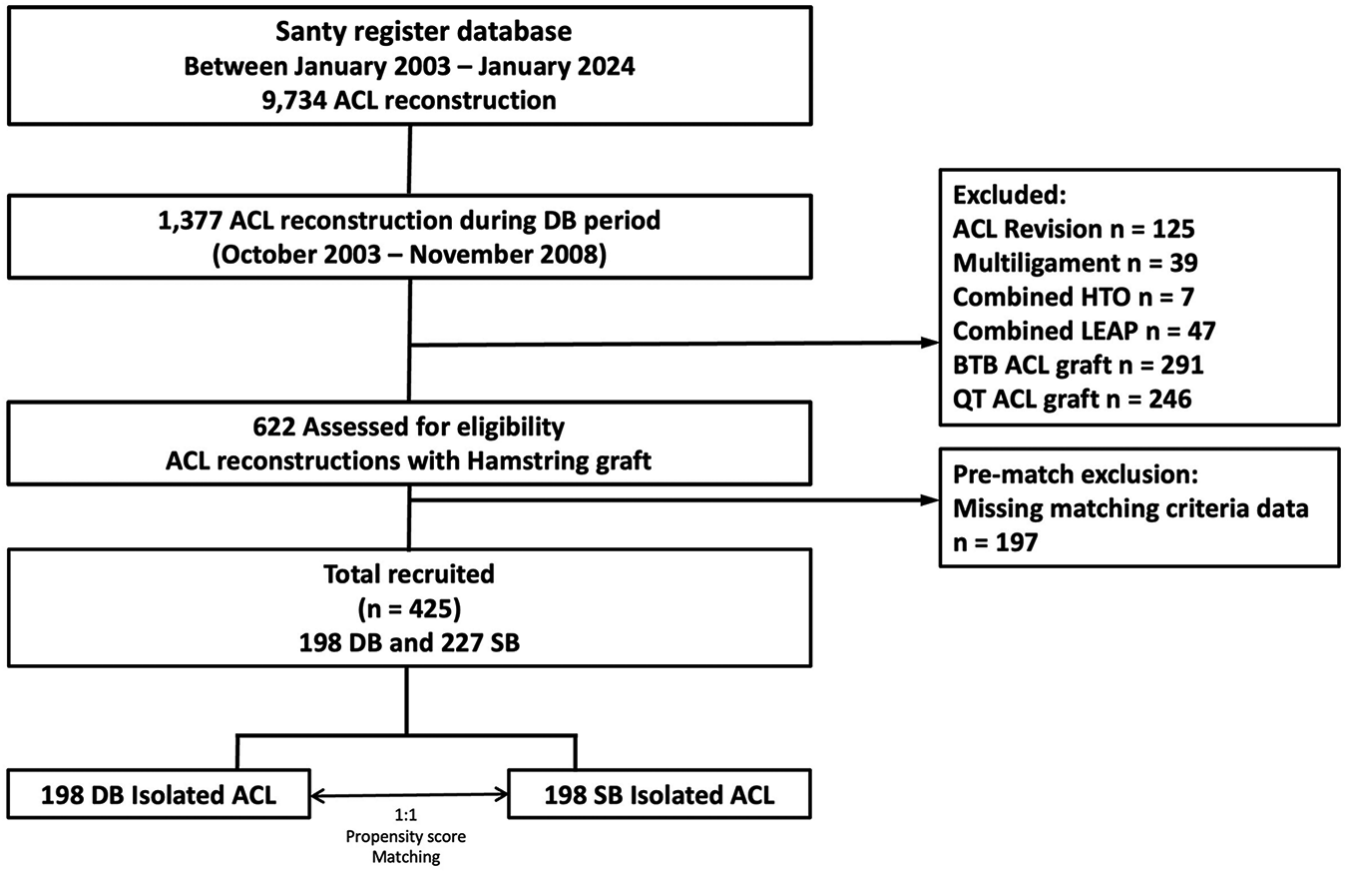
Study flowchart. ACL, anterior cruciate ligament; BTB, bone-tendon-bone; DB; double-bundle; HTO, high tibial osteotomy; LEAP, lateral extra-articular procedure; QT; quadriceps tendon, SB, single-bundle.

During the time frame in which the senior surgeon performed the DB technique, 1377 ACLRs were carried out. After applying the inclusion and exclusion criteria, the final population consisted of 425 patients. Each patient in the DB group was then paired with a single best-matched patient from the SB group using propensity matching (Figure 3).

A total of 198 patients were included in each group.Patient characteristics were comparable between the groups. The characteristics of the study population, stratified by group, are reported in Table 1.

**Table 1:** Characteristics of Study Population by Group, According to Criteria Used for Propensity Matching*^*a*^*

	SB ACLR (n = 198)	DB ACLR (n = 198)	*P* Value
Male sex	126 (63.6)	152 (76.8)	<.01
Age, y	29.9 ± 10.8	28.7 ± 9.1	.16
Body mass index, kg/m^2^	23.56 ± 3.01	24.03 ± 3.08	.08
Pivoting sports participation	187 (94.4)	183 (92.4)	.43
Contact sports participation	106 (53.5)	125 (63.1)	.05

a Data are presented as mean ± SD or n (%). ACLR, anterior cruciate ligament reconstruction; DB, double-bundle; SB, single-bundle.

Matching successfully achieved covariate balance between the groups, with all absolute standardized differences (ASDs) <0.25. The maximum ASDs for the analyzed variables were as follows: age (0.083), BMI (–0.028), sex (0.181), meniscal lesion (0.013), and Tegner activity score (–0.058).

### Clinical Outcomes

At a mean follow-up of 176.7 ± 7.7 months (range, 166-211 months), 40 patients (10.1%) experienced a graft rupture: 22 patients (11.1%) in the SB group and 18 patients (9.1%) in the DB group (*P* = .52). Also, 25 patients underwent subsequent surgical procedures, including cyclops syndrome, arthrolysis (*P* = .65), or secondary meniscectomy (Table 2). No significant differences were noted between the 2 groups in terms of reinterventions.

**Table 2:** Summary of Secondary Surgeries*^*a*^*

	SB ACLR (n = 198)	DB ACLR (n = 198)	*P* Value
Graft rupture	22 (11.1)	18 (9.1)	.52
Cyclops syndrome	5 (2.5)	7 (3.5)	.56
Arthrofibrosis	3 (1.5)	2 (1)	.65
Meniscal pathology	7 (3.5)	6 (3)	.76

a Data are presented as mean ± SD or n (%). ACLR, anterior cruciate ligament reconstruction; DB, double-bundle; SB, single-bundle.

At 1 year postoperatively, no significant differences were seen in anteroposterior side-to-side laxity difference between the groups. The mean side-to-side laxity difference using a Rolimeter was 0.4 ± 0.9 mm in the SB group and 0.6 ± 1.1 mm in the DB group (*P* = .11).

### Survivorship and Risk of Subsequent Surgery Analyses

**Figure 4.**
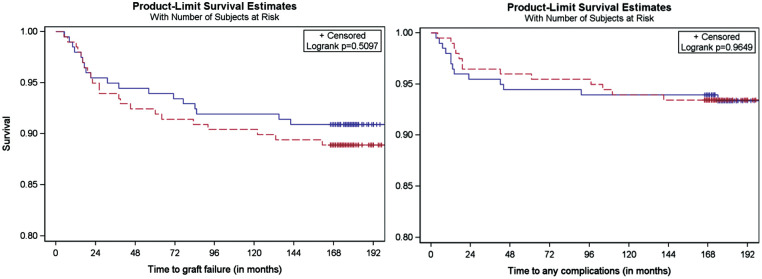
Left: Kaplan-Meier plot demonstrating the differences in graft survivorship between the single-bundle (red) and double-bundle (blue) techniques (11.1% vs 9.1%). No significant differences were found (*P* = .51). Right: Kaplan-Meier plot demonstrating time to any complication survivorship between the single-bundle (red) and double-bundle (blue) techniques (6.6% vs 6.6%). No significant differences were found in time to complications between both techniques (*P* = .96).

Kaplan-Meier analysis demonstrated no significant differences in graft survivorship or overall reoperation rate between the SB group and the DB group (Figure 4).

Multivariate Cox proportional hazards analysis showed no significant association between graft failure and surgical technique (HR, 0.857; 95% CI, 0.457-1.609; *P* = .63). In the adjusted model, which was stratified by matched pairs and adjusted for all risk factors, age, sex, BMI, time from injury to surgery, type of sport, preoperative laxity, and Tegner activity score were also not significantly associated with graft failure.

## Discussion

The main finding of the current study is that SB and DB ACLR techniques using hamstring autograft resulted in comparable long-term clinical outcomes, with no significant differences in graft survival, reoperation rates, or knee stability at a mean follow-up of >14 years.

Our results are also consistent with several other mid- to long-term studies that have shown that DB ACLRs, although theoretically advantageous for rotational control, have not consistently outperformed SB ACLRs in terms of clinical measures such as graft survival and postoperative stability. Recently, Balasingam etal^3^ published the clinical results of a prospective randomized study comparing DB and SB ACLRs at 10 years of follow-up.The authors found no significant differences between the groups in terms of clinical and radiographic outcomes, with both techniques demonstrating similar improvements from baseline in pivot-shift grade, knee laxity, Lachman test results, functional scores, and radiographic OA progression. Additionally, the same group reported the clinical and radiographic outcomes of this randomized controlled trial (RCT) at the 5-year follow-up.^9^ Similarly, they found that both techniques resulted in comparable improvements in knee stability, patient-reported outcome measures (PROMs), and hop test results, and no differences in OA rates were observed between groups, although a significant increase in OA was noted within the DB group over time. Nonetheless, another RCT comparing DB and SB ACLRs in 130 patients with at least 4 years of follow-up did not find radiographic OA progression to be significantly different.^22^ Equally, graft failure, PROMs, and stability outcomes (Lachman test, pivot-shift test, and stress radiography) were comparable between groups. These findings are consistent with the results of our current study.

Conversely, Järvelä etal^8^ published the results of an RCT comparing DB and SB ACLR at the 10-year follow-up and found significantly fewer graft failures in the DB group compared with the SB group.Among the 81 patients included in the analysis, there were no significant differences in pivot-shift test results, KT-1000 arthrometer measurements, knee scores, or OA rates between the groups. However, the same group published the 15-year follow-up results and found no statistically significant differences in graft failure rates, subjective assessments, knee stability, range of motion, or functional testing, similar to our findings.^20^ In another RCT, Muneta etal^14^ concluded that DB ACLR is superior to the SB technique with regard to anterior and rotational stability. However, there were no significant differences in range of motion, muscle strength, Lysholm scores, or subjective outcomes between the groups, suggesting that the DB reconstruction provides better stability but does not provide subjective improvements over the SB reconstruction.

Van Eck etal^27^ evaluated predictors of ACLR failure in 168 patients with DB and 38 patients with SB and identified younger age and early return to sport as the main predictors of graft failure in the DB group, whereas increased body mass index and younger age were associated with SB failure. However, in the current study, graft failure was not significantly associated with any of the measured factors, including age, BMI, sex, time from injury to surgery, participation in rotational or contact sports, concurrent meniscal injury, preoperative laxity, and Tegner activity score. None of these parameters showed significant differences between the DB and SB groups. While recent studies comparing graft failure rates have not extensively addressed patient-specific factors contributing to failure, Suomalainen etal^26^ suggested that rotational instability in the SB group may increase graft failure rates at the 2-year follow-up.

Specific complications of ACLR are well studied for individual techniques.^5,24^ There is, however, a lack of studies that compare the DB technique and SB technique with regard to complications or reoperations other than graft failure rates. In the current study, there was no significant difference between the DB and SB groups in relation to the number of complications.

Most of the trials comparing SB and DB ACLR have focused on postoperative laxity as the primary outcome. In our study, no significant difference in anterior laxity was observed between the groups, with a mean anteroposterior side-to-side laxity of 0.6 mm in the DB group and 0.4 mm in the SB group.Still, there is no clear consensus. For example, in a study of 328 patients with a 2-year follow-up, Kondo etal^11^ found significantly better postoperative anterior and rotational stability in the DB group, with a mean anterior laxity of 1.2 mm compared with 2.5 mm in the SB group.Similar findings have been reported in other studies.^6,7,10,18,28^ However, in a study including 113 patients, Park etal^18^ found no significant difference in anteroposterior laxity at 2 years, with findings similar to a recent RCT by Balasingam etal,^3^ who also found no significant differences in postoperative knee laxity between DB and SB techniques. These findings are consistent with our results and suggest that a clear superiority of the DB technique to the SB technique in managing postoperative laxity has not been proven.

The present study is not without limitations. First, this was a retrospective, nonrandomized, database registry-based study, which may introduce selection bias. However, to mitigate this risk, propensity score matching was used to balance key patient and clinical variables between the SB and DB groups. Second, all procedures were performed by a single high-volume surgeon, which may limit the generalizability of our findings. Although this helped maintain consistency in surgical technique and postoperative management, outcomes may differ in other surgical settings or with varying levels of surgical expertise. Similarly, return to sport was allowed at 4 months for nonpivoting sports, 6 months for pivoting noncontact sports, and 8 to 9 months for pivoting contact sports, which may differ from current protocols in many countries. Additionally, as a registry-based study, data collection was limited to the variables recorded in the database, which may have restricted the scope of analysis. Finally, this study focused primarily on objective measures (graft failure, reoperations, and side-to-side laxity) rather than a comprehensive assessment that includes PROMs. Despite these limitations, the relatively large cohort of patients and the use of robust statistical methods, including matched-pair analysis and stratified multivariate models, strengthen the validity of these findings.

## Conclusion

Our study showed that over a mean follow-up of >14 years, the SB and DB ACLR techniques using hamstring autograft showed similar clinical results in terms of graft survival, reoperation rates, and stability. These results suggest that both techniques offer comparable efficacy in the long term.
